# A new measure of node centrality on schedule-based space-time networks for the designation of spread potential

**DOI:** 10.1038/s41598-023-49723-9

**Published:** 2023-12-19

**Authors:** Dino Pitoski, Karlo Babić, Ana Meštrović

**Affiliations:** 1 Center for Artificial Intelligence and Cybersecurity, University of Rijeka, Rijeka, Croatia; 2 Faculty of Informatics and Digital Technologies, University of Rijeka, Rijeka, Croatia

**Keywords:** Engineering, Mathematics and computing

## Abstract

Node centrality is one of the most frequently revisited network theoretical concepts, which got many calculation method alternatives, each of them being conceived on different empirical or theoretical network abstractions. The vast majority of centrality measures produced up to date were conceived on static network abstractions (the so-called “snapshot” networks), which arguably are less realistic than dynamic (temporal) network abstractions. The new, temporal node centrality measure that we offer with this article, is based on an uncommon abstraction, of a space-time network derived from service schedules (timetables). The proposed measure was designed to rank nodes of a space-time network based on their spread or transmission potential, and was subsequently implemented on the network of sea ferry transportation derived from the aggregated schedules for sea ferry liner shipping services in Europe, as they occurred in the month of August, 2015. The main feature of our measure, named “the Spread Potential”, is the evaluation of the potential of a node in the network for transmitting disease, information (e.g. rumours or false news), as well as other phenomena, whichever support a space-time network abstraction from regular and scheduled services with some known carrying capacities. Such abstractions are, for instance, of the transportation networks (e.g. of airline or maritime shipping or the wider logistics (delivery) networks), networks of medical (hospital) services, educational (teaching) services, and virtually, of any other scheduled networked phenomenon. The article also offers the perspectives of the measure’s applicability on the non-scheduled space-time network abstractions.

## Introduction

Node centrality is arguably one of the most frequently re-evaluated concepts in Network Science. Many measures have been developed, and continue to emerge, having the common purpose of ranking nodes in terms of their relevance in a network, based on different network abstractions, derived from diverse empirical or theoretical data(^1–3^; to name only a few). In the real world, the networked behaviour is dynamic, which invites for a kind of network abstraction that incorporates timestamps in order to capture the nodes’ interactions as they unfold in real time. However, what commonly can be found in network science literature are static network abstractions, in which the interactions occurring at different points in time between unique node pairs in a wider time interval are subsumed to stand for network links (weights) per each pair. Most likely reasons that the space-time network abstractions are not largely represented in the literature are the lack of data (collection) resources to abstract such networks and the computer power needed to subsequently analyse them. While data appear to be everywhere, securing resources, both human and technological, to collect these in a systematic fashion for a credible abstraction of networks from data, is a much more demanding task. Yet, the way the networks get abstracted from data is critical for their subsequent assessment, as the abstractions on which the network measures get executed inherently determine the reliability and soundness of these measures’ designs, thereby their further usability by scientists, decision-makers and other potential beneficiaries^4^. (Note that, throughout this text, notwithstanding the possible theoretical differences between the terms, we will interchangeably use “space-time”, “temporal”, “dynamic”, “longitudinal”, “time-ordered”, and “time-varying” networks as terms designating one and the same construct.)

Upon the advocations for moving towards the more realistic, space-time network abstractions, and subsequently analyses of such networks, stands the fact that, in the act of aggregation of the interactions to form the links (weights) connecting the nodes over some time frame—a common practice producing the so called “snapshot” network abstractions—there is the risk that one may obtain incomplete, or even misleading, information on the actual connectivity (of a node, link, complete network, or its communities). This issue can be clarified by an example of a simple network with 3 nodes, *i*, *j*, and *k*; at each subsequent time point $t+m$, where *m* is some random interval within a wider time frame over which one counts the occurrences of the nodes’ connectedness, it could be that a connection has been established between, and only between, a different *i*, *j* pair. In the static/snapshot view of a selected wider time frame, which represents the sum of all link realizations over that frame, one will be considering a clique, although no complete connectedness of the network nodes occurred at any moment (or at least a “close-enough” moment) within the frame. Correcting for this issue is especially important with real-world network case examinations, such as the spread of disease; as one may wrongly conclude there was the potential for the transmission, although in reality there was none. As part of these network abstraction issues, and in particular the phenomena of the disease transmission, node centrality, which essentially is designed to outline by ranking the most influential spreaders in the network, is arguably the most important concept the network science should seek advances for.

Beyond the aforegiven theoretical-exemplary argument for advancing towards space-time network abstractions, there are some empirically supported arguments that push for these advances, which have recently emerged in applied network research, in the works of^4–6^. Analysing the phenomenon of human migration, and abstracting migration networks as static (with weights of links being the total counts of people migrating between any two human-settlement pairs over a one-year period), authors run across issues such as extreme weights on self-loops and high reciprocity, and demonstrate the hindering effect that these characteristics have on informativity of many of the established indicators and algorithms, when applied to these specific networks. Although the authors tried to deploy appropriate statistical inference tools to justify the required modifications (simplifications) of the analysed networks prior to the measures’ implementation—which modifications include the removal of looping edges before the application of network metrics (an ubiquitous practice in network science literature)—the otherwise straightforward applications of the indicators and algorithms on the static network abstractions, for the case of migration, were needed to be designated as “to be taken with reservations”. With space-time network abstractions, both the issue of self-loops and the issue of reciprocity, which certainly occur in other networks than that of migration, get resolved, and our methodology and further application of the developed indicator (“Methodology” and “Application: European scheduled freight ferry shipping”)—as just one, yet fundamental application—are offered to demonstrate how.

Besides the indicator methodology and application presented herewith, the studies dedicated to developing indicators for dynamic networks are overall quite scarce. The few works produced by the network science community on the topic are covered in “Related works”. Our unique measure is offered to join this small set of calculation designs, which might show as particularly useful in the analysis of the contemporary phenomena, given its use perspective of *spreading* that fits the real-world circumstances, with major ones being the spread of diseases such as Corona (COVID-19), or the spread of (fake) news throughout the social media and the World Wide Web. Our measure is adjusted for space-time network abstractions, while incorporating weights in the calculation.

In “Related works”, we promote the literature that we found to be most related to our work. In “Methodology”, we explain the theoretical model for the calculation of our node centrality measure for space-time networks—the “Spread Potential”, which we subsequently apply on the space-time network of the scheduled sea-ferry transport in Europe; in “Application: European scheduled freight ferry shipping”. We discuss our findings, including the notions on how to extend the indicator methodology to the non-scheduled space-time network applications, and on the offered alternative uses of our indicator, in “Discussion”.

## Related works

Network science constantly produces new measures to capture various network features, but node centrality continues to be one of the most intriguing concepts. This can be established by looking only at the amount of studies dedicated to designing new node centrality indicators as compared to other network indicators (e.g. indicators for links’, or communities’, assessment). The latest, more recognized examples of node centrality design (in terms of the rise in these works’ citations over a very short period from their publishing), include^3^,^7–10^. However, when assessing in more depth the overall literature dedicated to developing these measures, one can clearly spot the scarcity of node centrality calculation designs offered for temporal networks, which brings back to the issue of uncommonness of temporal network abstractions in research in general.

Behind this short section, in which we touch upon only some of the related works, is an extensive investigation into the Google Scholar bibliographic database, which we performed using the tool Publish or Perish^11^, in order to browse for as many as possible related titles using the primary keyphrase “node centrality” with secondary keyphrase variants such as “temporal networks”, “time-varying networks”, “dynamic networks” and various other variants for the titling of the investigated concept. Our search was performed on 13th December, 2022. We have examined the abstracts (or, when required, introductions) of the outlined works, to check whether they were focused on designing an indicator for temporal node centrality. References sections of the examined studies were also checked to seek for potentially omitted titles. Suggestions on additional relevant works came from other experts in the field as well, such as also the reviewers of this work. Overall, we were able to distinguish the titles that we address below.

The strand of research that has produced centrality measures for dynamic (temporal) networks most likely begins with^12^, while most of the previously developed measures were covered by the review of^13^; namely^14–35^. Our work ties perhaps most strongly to the attempts to adapt the measure of closeness centrality to fit the dynamic perspective, i.e. *temporal closeness*, as offered by^34,35^. Similar perspective has recently been taken by^36–38^. Here we do not cover each and exact previous measure design, methodology and application (as most of these have already been covered by the aforementioned^13^), but only emphasize the main features by which our measure differs from any of the previous ones, as follows.

Our measure, and its background methodology, is different from any of the previous measures/methodologies in the way that it does not *at all* require parsing of the wider time frame of phenomena observation into intervals of the same length for the calculation, and that it observes *all* possible paths; paths extending over the whole observational time frame. Moreover, it incorporates link weights in the temporal centrality calculation, which, to our knowledge, has not been included in any of the previous measure designs. Unlike in the previous works where the centrality is assigned to the node as person (an “agent”, see^37^), our measure is assigned to the node as a spatial unit, which spatial unit is essentially designated as more/less riskier location for the spread of the researched phenomenon (disease, information, or other). The following display of our measure’s methodology should clarify the differences for the readers who might go into comparing ours with any of the previously offered. Also, in “Application: European scheduled freight ferry shipping” we provide a comparison of our measure results and those obtained using the methodology developed in the aforementioned^35^.

## Methodology

The calculation methodology of our indicator, the “Spread Potential”, is conceived on the network abstraction of a phenomenon that “operates” according to a timetable; i.e. a scheduled network. This scheduled network matches the following real-world network application (“Application: European scheduled freight ferry shipping”), and the scheduled aspect, in parallel, facilitates the elaboration of the mathematical model. The generalization to unscheduled applications too is possible, as we discuss in “Discussion”.

**Figure 1:**
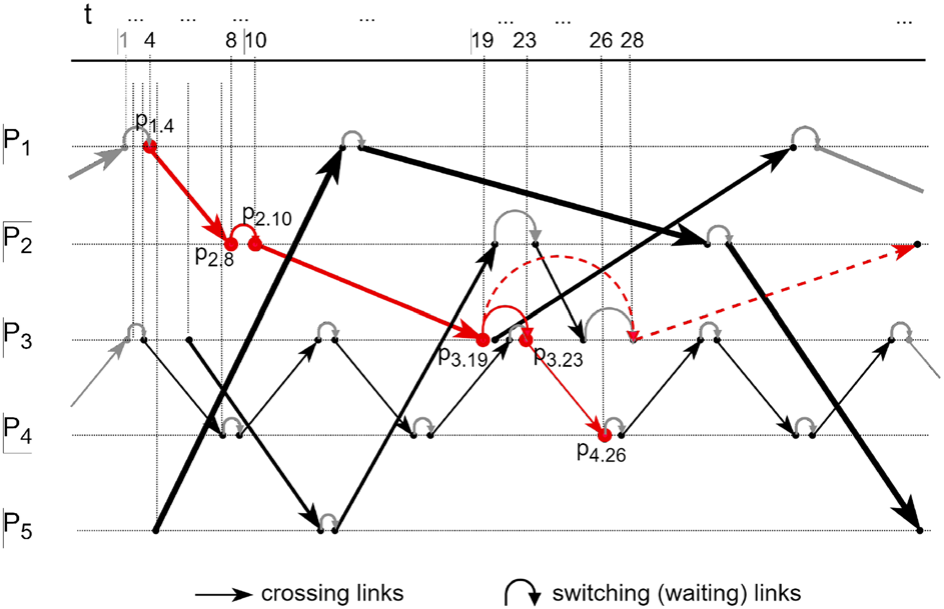
Temporal network for a hypothetical service schedule.

In Fig. 1, we provide a hypothetical temporal network based on some service schedule, where we consider *P*—ports—as the entering/exiting locations of the disease/information/other-item’s transmission.

With reference to the figure; let *P* be the set of all ports $P_k$ appearing in the consolidated schedules of the service observed. Furthermore, let *V* be the set of all nodes $p_{k.i}$, which stand for the positions, or actions, of entering or exiting the port $P_k$ at precise time $t_i$, as deduced from the consolidated schedules; in further text also *positions*, or *nodes*.

Now, consider a directed graph $G = (V, L)$, with $L=\left\{ L_{k.i,l.j}\right\} \cup \left\{ L_{k.i,k.j}\right\}$, where:

- $L_{k.i,l.j}$ is the set of links $l_{k.i,l.j}$ that connect positions $p_{k.i}$ and $p_{l.j}$ which regard different locations (ports) $P_k$ and $P_l$ exited and entered, respectively, at times $t_i$ and $t_j$, which can be derived from the consolidated service schedules (e.g. departures and arrivals); in further text: *crossing* links, and
- $L_{k.i,k.j}$ is the set of links $l_{k.i,k.j}$ that connect any two subsequent positions $p_{k.i}$ and $p_{k.j}$, which regard the same location (port) $P_k$, and which links exist if $T_{L}<(t_j-t_i)<T_{U}$. In further text we refer to these as *switching* or *waiting* links.

The latter lower ($T_{L}$) and upper ($T_{U}$) bound of the time interval for accounting for the switching links is arbitrary, and should be adjusted with respect to a specific problem application (e.g. disease transmission, the spread of rumours, etc.). These time bounds are crucial in establishing the potential paths in the network, and essentially in the network abstraction as a whole. Pointing back to Fig. 1, it matters a lot how we set these thresholds, especially the upper bounds. For example, we might allow a switch from position $P_{3.19}$ to position $P_{3.28}$ by extending the bounds, thus obtaining another path in addition (dashed), increasing the connectivity of the network in general, which has consequence on the application of our, as well as any other, temporal network measure. We return to discussing the rationale for the selection of the time bounds later in the section, as well as when delivering our case application (“Application: European scheduled freight ferry shipping”) (Fig. 1).

After defining the links, assign to each link in the set $L_{k.i,l.j}$ a weight equal to the carrying capacity of the means by which the scheduled service is executed (for instance, aircraft capacity, measured by the number of seats of an aircraft used in the specific service, in the case of airline transportation scheduled network). To each link in the set $L_{k.i,k.j}$ attach a minimum positive weight. (The weight that is placed onto the switching link should be approaching zero as to ensure link’s existence without affecting the results in the path sum-of-weights as described later in the text.). Let *W* denote the set of according links’ weights $w_{k.i,l.j}$, as we continue to observe a weighted directed graph, $G_w=(V, L, W)$.

In the established weighted directed graph, for each node (position) observe all possible outgoing paths from that (evaluated) position to other subsequent positions in the graph. Let ${{ex}_{m}}({p_{k.i}}) \in {EX}({p_{k.i}})$, designate the (set of) paths outgoing from an evaluated node $p_{k.i}$ to all other $p_{l.j}$ for which $j>i$, where an ${{ex}_{m}}$ may not include the switching link $l_{k.i,k.j}$ touching the same $P_k$ as the one pertaining to the evaluated position, or the position already traversed in the path. In other words, a path cannot begin with a switching link, nor can it pass a port more than once (which includes not returning to the starting port).

Now, the calculation for the spread potential (*SP*) of a port $P_k$ is defined by the following Eq. (1):

1 $$\begin{aligned} SP(P_k)=\sum _{i} \sum _{{l.j}\in {EX}({p_{k.i}})} {w_{k.i,l.j}} \end{aligned}$$

In the equation, keep note that while naturally $i \ne j$ (as the notation refers to consecutive time points), *k* can be equal to *l*, enabling accounting for the (infinitesimally small) weights on switching links along the observed paths.

To understand better the above formulation, observe the Fig. 1 at, for example, position $p_{1.4}$ at the upper left. One (simplest) potential path stemming from the position is ${{ex}_{1}}({p_{1.4}})$: $p_{1.4}$ $\rightarrow$ $p_{2.8}$. The second potential path ${{ex}_{2}}({p_{1.4}})$ is $p_{1.4}$ $\rightarrow$ $p_{2.8}$ $\rightarrow$ $p_{2.10}$ $\rightarrow$ $p_{3.19}$, if the switch $p_{2.8}$ $\rightarrow$ $p_{2.10}$ is within the decided time bounds $T_{L}$ and $T_{U}$. The third possible path from the currently evaluated $p_{1.4}$ is ${{ex}_{3}}({p_{1.4}})$ is $p_{1.4}$ $\rightarrow$ $p_{2.8}$ $\rightarrow$ $p_{2.10}$ $\rightarrow$ $p_{3.19}$ $\rightarrow$ $p_{3.23}$ $\rightarrow$ $p_{4.26}$, if both the switch $p_{2.8}$ $\rightarrow$ $p_{2.10}$ and $p_{3.19}$ $\rightarrow$ $p_{3.23}$ are executable within the decided time bounds. Subsequently, in the same manner, one establishes each path starting from all positions $p_{1.i}$, effectively summing the weights on each of these paths’ links, proceeding until all the paths outgoing from the positions pertaining to port $P_1$ are evaluated. The sum over all particular position-level values per port $P_1$ produces the final value of the spread potential of the port $P_1$.

To understand better the rationale behind the formulation, imagine a situation where an infected person enters the system at any given time in the space-time network abstracted from schedules, e.g. as in one previous relation, a passenger loading an airplane of capacity *w* at port $P_{k}$. That person may infect other people in the aircraft (or at an airport), as long as s/he stays in her micro-environment (i.e. the aircraft or a port) for a precised amount of time. Capacities, or link weights, in this case, are a proxy for the size (i.e. potential) of the disease spread, as long as the person remains on the path to another port. The sum of all potentials for the spread considering an infected person gets “inserted” in a specific port at any given moment will designate the total spreading potential (probability) of/from that particular port. Note that the capacities can be adjusted using some case-specific parameters, for this instance, the basic reproduction number ($R_0$)^39^, while the application-suitable time bounds to allow for switching links within a path can also be selected accordingly. Note also that we do not suggest using that one aircraft’s capacity as proxy for the spread when keeping at ports (i.e. on switching links), as the generally unknown amount of aircrafts at that port in the same time interval are already included in the crossing capacities with other port-positions evaluations. Concerning the feasible time bounds to make a switch in the same port to proceed to another, thus maintaining the path, for the airline networks example these time bounds may be set to, e.g. 1.5–5 h between the time of landing and the time of flying out of the same port. The idea is that the passenger may *manage* to switch to the flight to another port within 1.5 h, while would *be willing* to switch (wait) for the flight to another port for 5 h, thus continuing infecting passengers on the subsequent trip. Further clarifications on the rationale for the measure are provided along with the application in the sequel.

## Application: European scheduled freight ferry shipping

In this section, we analyse the performance of the developed centrality indicator on the example of the scheduled shipping services for the scheduled freight ferry transport in European wider region. The region comprises sea ports located in the European Union (EU), as well as in non-EU countries such as Norway, Russia, Turkey, or the countries of the African Mediterranean. The services comprise transportation by Ro-Ro and Ro-Pax vessels; see the definitions under “RORO variations” at https://en.wikipedia.org/wiki/Roll-on/roll-off. The shipping schedules data have been collected manually from the websites of 18 freight ferry service providers for the period of two weeks in the month of August, 2015. These providers offered over 200 different routes altogether, spanning across more than 100 ports. The dataset, comprising consolidated schedules, including web-links from which the schedules were retrieved, is available as Supplementary Data^40^. Data on the ships’ capacities deployed on each route (path) were not available for most routes; we have instead collected the data on maritime distances (in nautical miles) between ports ($d_{k.,l.}$), using some free online web services, such as https://sea-distances.org/. These distances were subsequently deployed for the link-weight approximation; $w_{k.i,l.j}=d_{k.,l.}$, which approximation is the alternative to no-weight link evaluation in the basic version of the indicator; $w_{k.i,l.j}=1$. The logic behind the distance-based approximation follows the consideration that, in transportation, it is generally valid that for the longer-distance voyages larger vessels (/means of transport) are engaged, in order to ensure the scale economies. This assumption is admittedly relatively weak, and spurred by a lack of alternatives in terms of the data collection, however, it may be viable in the alternative use cases of our indicator application, which we discuss in “Discussion”. In terms of the time bounds, for this initial application we have determined 30 min as the lower time bound, and 180 min as the upper bound, with the idea that the passenger can feasibly switch to a next voyage from the same arrival port in 30 min, while be willing to make that switch within maximum 180 min.

The pseudocode for the spread potential algorithm with general weight notation in the weighted space-time network abstraction is provided as Algorithm 1 below, followed by the results for both the binary and the weighted abstraction; in Table 1. Python code for the algorithm is available on GitHub: https://github.com/karlo-babic/spread-potential. An interactive visualization of the analysed network is available at: http://bit.ly/3ENbTme.

**Algorithm 1:**
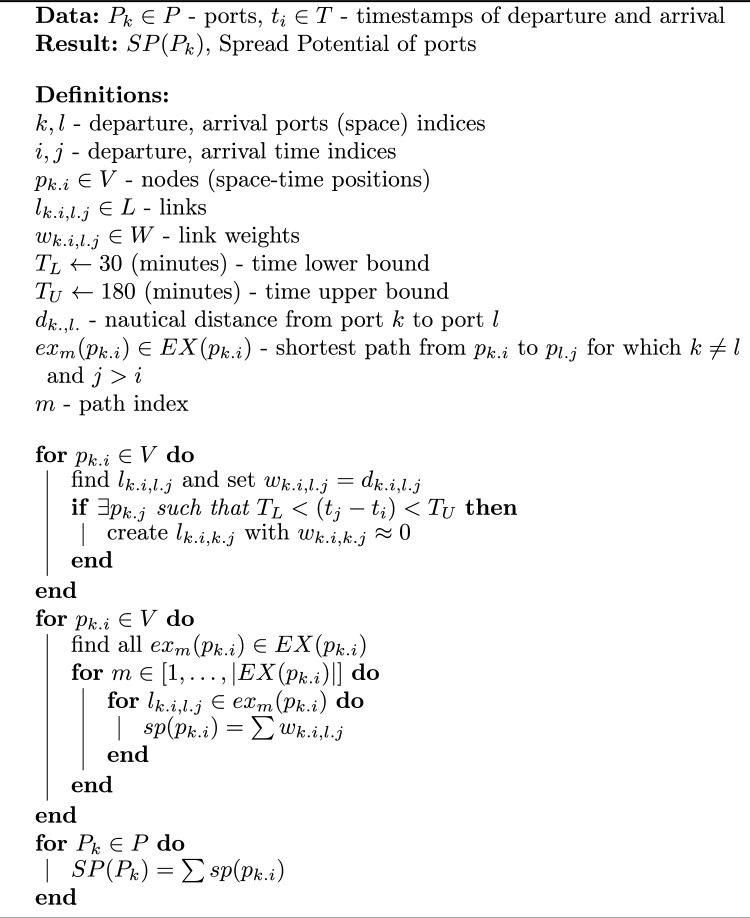
The Spread Potential of ports (weighted version).

**Table 1:** Node centrality rankings; spread potential vs. selected node centrality measures for static networks (top 30 ports).

${{{Port}}}$	${{ {SP-B}}}$	${{ {NS- B}}}$	${{ {NOS-B}}}$	${{ {PR-B}}}$	${{ {SP-W}}}$	${{ {NS-W}}}$	${{ {NOS-W}}}$	${{ {PR-W}}}$
	Spread potential (binary)	Node strength (static from binary)	Node out~strength (static from binary)	PageRank (static from binary)	Spread potential (weighted)	Node strength (static from weighted)	Node out~strength (static from weighted)	Pagerank (static from weighted
${{{CALAIS}}}$	1572	846	444	0.008472	56,424	23,688	12,432	0.007195
DUNKERQUE	984	304	152	0.003956	40,048	15,200	7600	0.005252
${{{DOVER}}}$	554	1150	554	0.011783	18,856	38,888	18,856	0.011801
${{{ROSTOCK}}}$	537	183	90	0.013864	71,435	26,322	11,950	0.013952
${{{LARNE}}}$	456	184	92	0.00688	31,756	6808	3404	0.004219
${{{TALLINN}}}$	438	154	76	0.010114	99,664	7700	3800	0.003574
${{{TRELLEBORG}}}$	352	370	181	0.025732	49,050	36,281	17,700	0.017961
${{{BELFAST}}}$	342	276	138	0.010981	31,185	27,224	13,612	0.011591
${{{CAIRNRYAN}}}$	330	344	172	0.012467	27,942	14,808	7404	0.007694
${{{HELSINKI}}}$	302	243	126	0.016918	90,252	58,071	30,946	0.022045
${{{YSTAD}}}$	298	162	80	0.009274	35,320	16,848	8320	0.007734
${{{HOLYHEAD}}}$	254	260	130	0.009802	39,390	26,780	13,390	0.0073
${{{KAPELLSKAR}}}$	254	186	92	0.010692	17,472	11,888	5820	0.007683
${{{LUBECK}}}$	248	240	122	0.021778	55,199	69,064	34,116	0.029884
${{{MARIEHAMN}}}$	242	376	188	0.020179	22,852	31,768	15,884	0.016109
${{{LIVERPOOL}}}$	235	140	70	0.005889	32,655	22,488	11,244	0.007917
${{{MALMO}}}$	230	76	38	0.006975	38,647	10,640	5320	0.005173
${{{SWINOUJSCIE}}}$	209	269	135	0.015625	24,004	27,869	13,987	0.012584
${{{PATRAS}}}$	204	88	52	0.00743	41,478	16,462	11,566	0.003865
${{{DUBLIN}}}$	192	356	180	0.013997	32,842	52,310	27,934	0.014943
${{{TURKU}}}$	192	112	56	0.00656	22,882	16,692	7828	0.00857
${{{FREDERIKSHAVN}}}$	186	166	82	0.014866	34,440	11,158	5530	0.005757
${{{ROTTERDAM}}}$	186	296	142	0.028269	45,614	63,772	28,850	0.027586
${{{HARWICH}}}$	180	88	40	0.009333	37,952	13,288	6040	0.007103
${{{IMMINGHAM}}}$	178	148	82	0.013156	52,662	54,734	29,776	0.02002
${{{GOTHENBURG}}}$	142	251	126	0.022578	35,954	59,013	29,260	0.021834
${{{STOCKHOLM}}}$	142	84	42	0.005041	11,162	6552	3276	0.004036
${{{FELIXTOWE}}}$	136	64	32	0.006626	29,968	9664	4832	0.00514
${{{IGOUMENITSA}}}$	136	188	96	0.019511	31,970	45,448	23,154	0.014758
${{{HEYSHAM}}}$	133	48	24	0.002834	13,449	7392	3696	0.003887

In Table 1, in the leftmost numeric column (SP-B) we provide the spread potential (SP) values for the top-30 (of, in total, 125) ports, sorted in descending order by SP values when applied in a binary network abstraction, that is, in which all weights on crossing links were set to equal 1 before algorithm’s execution. The full ranking of ports is available as the Supplementary Data^40^. Column SP-W shows the results of our algorithm run on a weighted network abstraction, in which the distance-proxied capacities were assigned on the crossing links.

We complement these rankings with rankings obtained by applying some additional measures designed for evaluating node centrality in static networks, which two we deemed as most comparable with our indicator: node strength (NS) and PageRank (PR), conceptualized in^41,42^, respectively. We run the measures on the static network abstraction in both the binary and the weighted setting. (We concluded that the application of the two comparable measures was not feasible for implementation on the temporal network abstraction. Essentially, our observations of only their methodologies led us to the conclusion that both NS and PR should produce the same result as when applied on the static abstraction.) In both settings, we sum all link realizations in the observed time frame per route, with difference being that in the binary setting all link realizations have the value of 1, while in the weighted setting all link realizations have the value equal to the maritime distance between the adjacent ports ($w_{k.,l.}$). For both the binary and the weighted version, we marked out separately the outward strength (NOS). Node strength is chosen as being the most intuitive and widely used measure for static weighted networks, essentially reflecting ports’ throughput; the in-node build-up of capacity that is incoming, or that is set for further distribution (spread) from the node to other *directly* connected nodes in the network. Outward, or out- strength, is the portion of this capacity specifically forwarded to the first-next connected ports in the system, and the same forwarding (spreading) perspective is, in a way, taken in our calculation methodology, though ours covering the *indirect* (or the so-called “neighbours-of-neighbours”) connectedness. PageRank is chosen for comparison as having been essentialized on that indirect connectivity, and consequently the potential of a particular node’s influence in the network, thus reflecting (that is to say, reciprocating) the main feature(s) of our measure.

**Figure 2:**
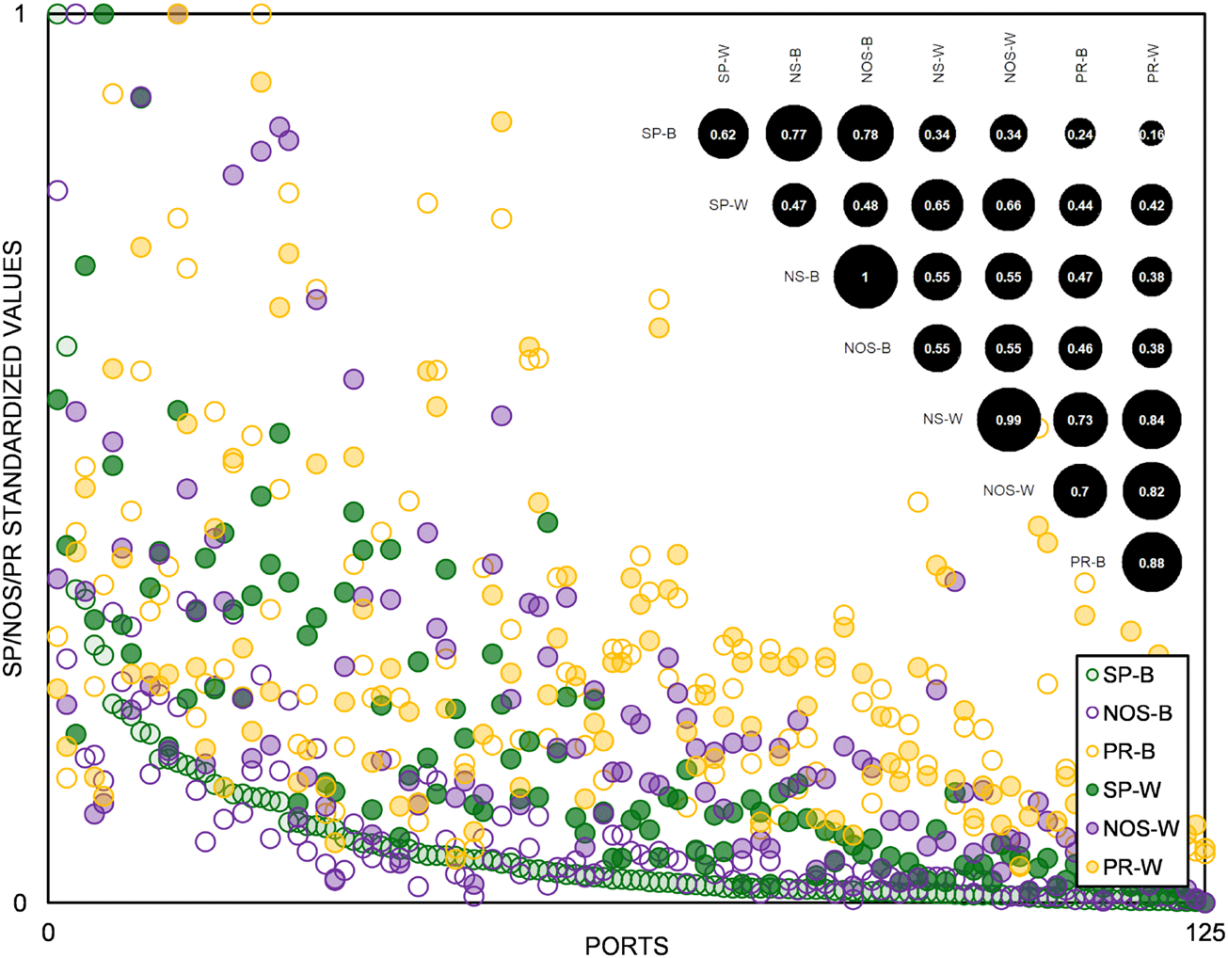
Correlations SP vs. selected static node centrality measures. Abbreviations in this figure match the headings of Table 1. Values for SP/NOS/PR are standardized dividing by maximum value from each indicator value set. Correlations (top right) are measured using Pearson correlation coefficient^43^.

In Fig. 2, we show the cross-correlations for all of the aforementioned indicators, along with the scatterplot with standardized indicator values for SP, NOS and PR in both binary (-B) and weighted (-W) version. Our indicator correlates relatively strongly with node strength when correlation is measured on values obtained from the application of both algorithms on binary network abstractions, and correlates relatively weakly with the same measure for values obtained in the weighted network setting. The correlations between the spread potential and PageRank tend towards an opposite direction; there is some correlation present in the weighted network setting, while much lower correlations between the same two concepts are obtained in the binary network setting. Correlation between the spread potential values calculated from its application on the binary network abstraction and those obtained when applying the same on the weighted abstraction is also relatively strong. The assessment of these correlations is not enough to reliably discuss on wider implications, yet the traced positive and strong correlations between the Spread Potential and some of the most widely used centrality metrics to some extent warrants the feasibility of our concept.

### Comparisons with other temporal network metrics

At this point we make a comparison with two of the related temporal network metrics, which stem from one of the first works in the field to upgrade centrality metrics to suit temporal network abstractions; namely *temporal degree (TD)* and *temporal closeness (TC)* of^35^.

It is important to note, and this returns to the differentiation highlighted in “Related works”, that the shipping schedules data that we have gathered, and that we abstract the temporal network from, are very different than the data used to develop the concept (as well as to run the metrics) in the aforementioned paper. Our data is on shipping schedules with nodes being tied to spatial units, while the application in^35^ involves mobile device collocations tied to persons. The most problematic part in the update to our network abstraction to suit the application of the two metrics is the decision on how to parse the whole observation interval of 14 days into equal shorter time intervals, as the concept imposes. For this case application, we have parsed the interval into days, which we believed is to be most sensible as most of the ships direct trips, even the shorter ones, take well over an hour, while can last for days.

We have run the two measures as in their original (binary) formulation, but we also made an extension to the metrics to incorporate link weights (TD-W and TC-W), in the generalization as proposed by^44^, which also is referred to in^35^. Our results (indicator rankings) are provided along with the previously discussed static metrics in the Supplementary material^40^ with the according headers (TD-B, TC-B, TD-W, TC-W).

In terms of the correlations, and firstly for the degree, the (Pearson) correlations of our indicators in both binary and weighted settings are roughly the same as what has been traced when comparing indicators calculated on static networks; $\rho$(SP-B, TD-B) $\approx 0.76$, $\rho$(SP-W, TD-W) $\approx 0.61$. This goes along well with the suggestions by the measure creators that “the normalized temporal degree is the same as the average value of the node’s degree in the time series of snapshot graphs”^35^ (pg 3).

However, with temporal closeness, which should be more comparable with our measure given that it bases on the shortest paths on an expanding set of intervals, correlation values turn out to be very low; $\rho$(SP-B, TC-B) $\approx -0.07$, $\rho$(SP-W, TC-W) $\approx -0.15$. This negative correlation in the weighted setting can be attributable to the fact that the temporal closeness, same as classical static network closeness centrality, takes inverse of links (weights) in the calculation, whereas our measure does not. However, the very low correlation in general clearly points towards the importance of a proper and unique network abstraction prior to the application of different temporal metrics and the reliable comparisons of the results.

An encouraging fact for us is that our measure correlates relatively well with some of the most adopted static network measures. Yet, as previously concluded, the assessment of these correlations is not enough to reliably concur on the wider implications. A thorough review and a comparative analysis with applications to an unique dataset and preferably a unique network abstraction that stems from this dataset, might be helpful in getting close to assessing the differences between the relatively small number of algorithms. A replica of the study by^45^, who assess community detection algorithms for static networks, might be useful for temporal node centralities, as well as potentially other network indicators, uncovering the “ground truth of networks” (here referring to the important philosophical discussions in the cited work). The indicators evaluated in such review should certainly include the more recent ones, such as^37,38^, as these arguably incorporate all previous knowledge in the field.

At the moment the number of indicators offered for temporal networks is still quite low. Given that fact, and given the novelty of our network abstraction and the offered algorithm code for free usage, we believe this work adds substantially to the scarce but important literature domain. More on the benefits of the measure and ideas for extensions follows as we close this paper.

## Discussion

The specific choice of a centrality indicator for the evaluation of the importance of a node in a network is arguably always an arbitrary one, and it has a lot to do with the application domain, as well as with how the analysed network has been abstracted from data in particular research cases. The benefits of a particular measure are perceivable only after testing the measure’s performance in a particular real-world setting; in our case, the aforementioned tests would have to involve inserting the disease, information, or any particular transmissible phenomena into specific nodes separately on different scheduled networks, and verify whether the real spread (measured by, e.g., the number of people catching the disease/information/etc.), when seeded at various nodes, correlates with the spread potential values of the same nodes. It is needless to say that undertaking such tests might be impracticable, if not unethical.

The spread potential conceived in this article is strongly tied to the notion of schedulability. In addition to the above thoughts on the feasibility-testing, if the execution of the algorithm would be done on an unscheduled space-time network, collecting the data needed for the abstraction of such a network would be even more demanding. Due to memory and processing issues, one may need to collect data for many shorter intervals within an evaluated wider time frame of the analysed networked phenomenon to be confident on the measure’s effectiveness. For example, if the transportation network, as the one analysed in this article, changes significantly over time or does not work on a scheduled basis, one should calculate the Spread Potential on several (representative-) sampled temporal networks abstracted from data for the relative time periods. This may not only be time/memory/computer-power consuming when it comes to the indicator calculation, but the data to abstract the network might be unavailable as the phenomenon’s dinamicity may make them unattainable/not feasible for collection. Nevertheless, for some networked phenomena, such as the spread of information via social media, where timestamps of information transmission between users are equivalent to the *positions* as defined in this article, the data to draw dynamic networks from, are almost readily available.

Next feasible step, thus, in advancing our developed measure, would be to run the algorithm on the dynamic network abstracted from a social media networks’ data. As a concrete example, one can observe the network of Twitter users and calculate the spread potential of each user to evaluate his or her influence in the network, or the impact s/he may have on the spread of information, and perhaps most desirably the impact s/he may have when it comes to the spread of “negative” information, such as false news. In the analogy with our application, the social media users’ accounts would be ports. The spread influence can be traced per all possible (re)tweets, or, one can observe the subnetworks of (re)tweeting, which subnetworks might be based on tweets falling into specific topic categories. Tracing these latter can be aided by semantic technologies such as keyword extraction, which, again, has been looked to be founded upon centrality metrics^46^. Simultaneously inserting some information to the users with higher, mid-range and lower spread potential and comparing with the size or the speed of contagion might be the least unfeasible experiment to test the measure validity, save the ethical considerations. In the forthcoming^47^ one will be able to find more elaborate discussions of the proposed approaches.

In regards to the above notion, it should be mentioned that our measure is envisaged on some partially tangible infrastructure (vessels, aircrafts, roads, corridors, etc.). In the case of social media networks, as opposed to transportation networks that we analysed and conceived our indicator on, there is no actual infrastructure connecting the users, except, perhaps, in the background, that of the internet. In that sense, the feasibility for the measure might be hindered with the measure obtaining an even more pronounced probabilistic (“potential”) character, as strongly depending on the inclusion of *all* possible interactions between users, and selecting sensibly, yet still arbitrarily, the representative intervals for temporal network abstractions before the algorithm’s executions.

Notwithstanding its yet unverified performance, and coming back to the arguments raised in the introduction to this paper, we believe our measure’s concept is significant ultimately due to the network abstraction on which it is conceived, which takes into account indirect connections as they *realistically* occur, in space and time. In that sense, although we can accept any critique regarding our “theorizing”-based methodology, our measure might be evaluated positively at least due to its ensuring that the actual indirect connections are included in the indicator calculation; unlike is the case with the standardly applied eigen-centrality measures such as PageRank or HITS algortihm^48^, which simply do not ensure the same in the real-world network applications where they are used (and so very often).

Lastly, we would like to point to an alternative potential use of our indicator, which should be welcomed by the policymakers and scientists engaged in research dedicated to developing connectivity indices for policy monitoring and control, in the domain of transportation, and some other domains, such as logistics, as well. In transportation, the policymakers’ initiatives for developing indicators for the evaluation of connectedness of particular nodes in transportation systems are decades old; most notable examples of such initiatives are the UNCTAD’s Liner Shipping Connectivity Index (LSCI)^49^, or the World Bank’s Air Connectivity Index^50^. Numerous subsequent initiatives and scientific attempts of designing indicators to evaluate node connectivity in transportation networks—especially those promoting the evaluation of port (/location) connectedness instead of country connectedness for a more fine-grained observation—followed, and continue to this date. For a review of connectivity indicators in maritime, as well as other transportation applications, which have emerged from both the political and scientific strands of literature, see^51^. These former attempts have been criticized for being simplistic and based only on the local information at each port^52^. Our connectivity indicator is directly offered as a desired enhancement.

### Supplementary Information

Supplementary Information.

## Data Availability

The datasets generated and/or analysed during the current study are available in the Figshare repository referred to and cited in the document as Supplementary Data^40^.
