# Supplementary figures and images for: A new measure of node centrality on schedule-based space-time networks for the designation of spread potential
Source: Sci Rep. 2023 Dec 19;13:22561. doi: 10.1038/s41598-023-49723-9 (PMC10728106; doi:10.1038/s41598-023-49723-9)

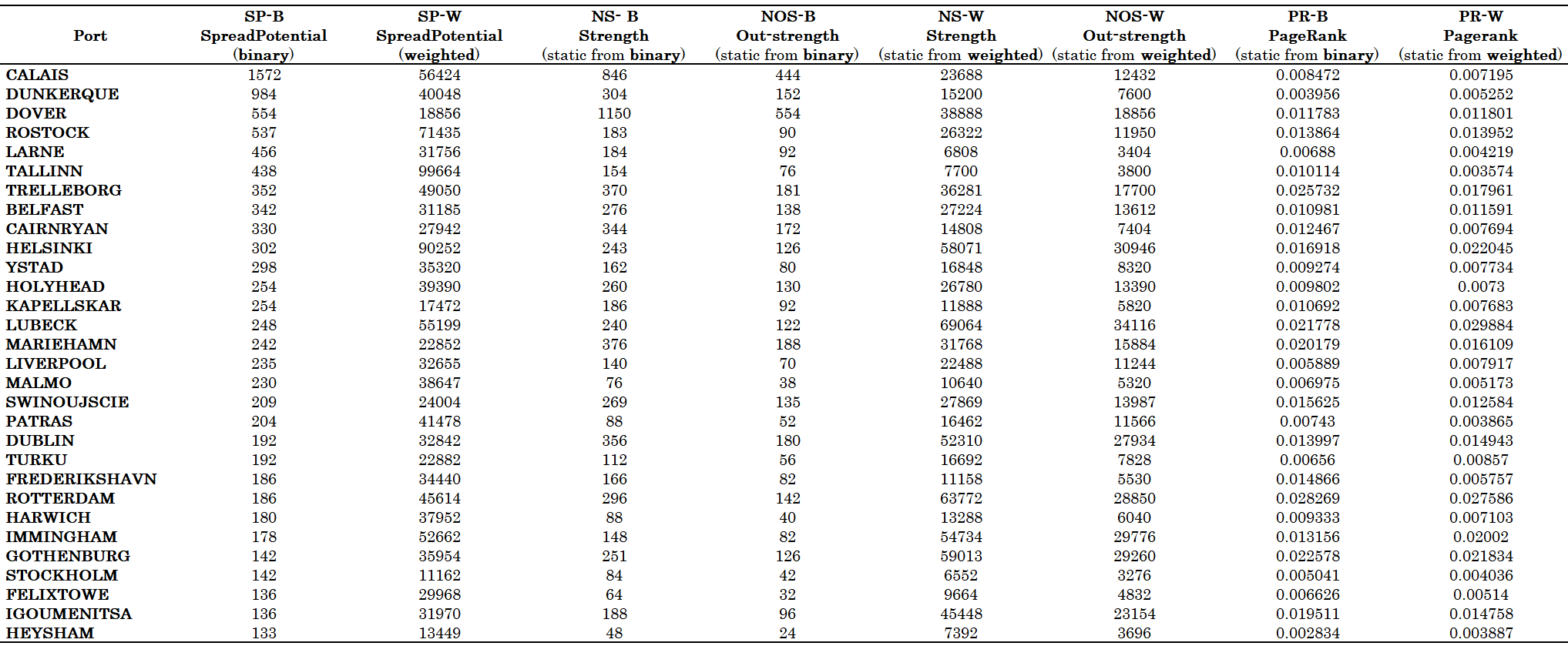

Supplement: Supplementary file 1 — Supplementary Information. [file 41598_2023_49723_MOESM1_ESM.zip › Ranking.png]

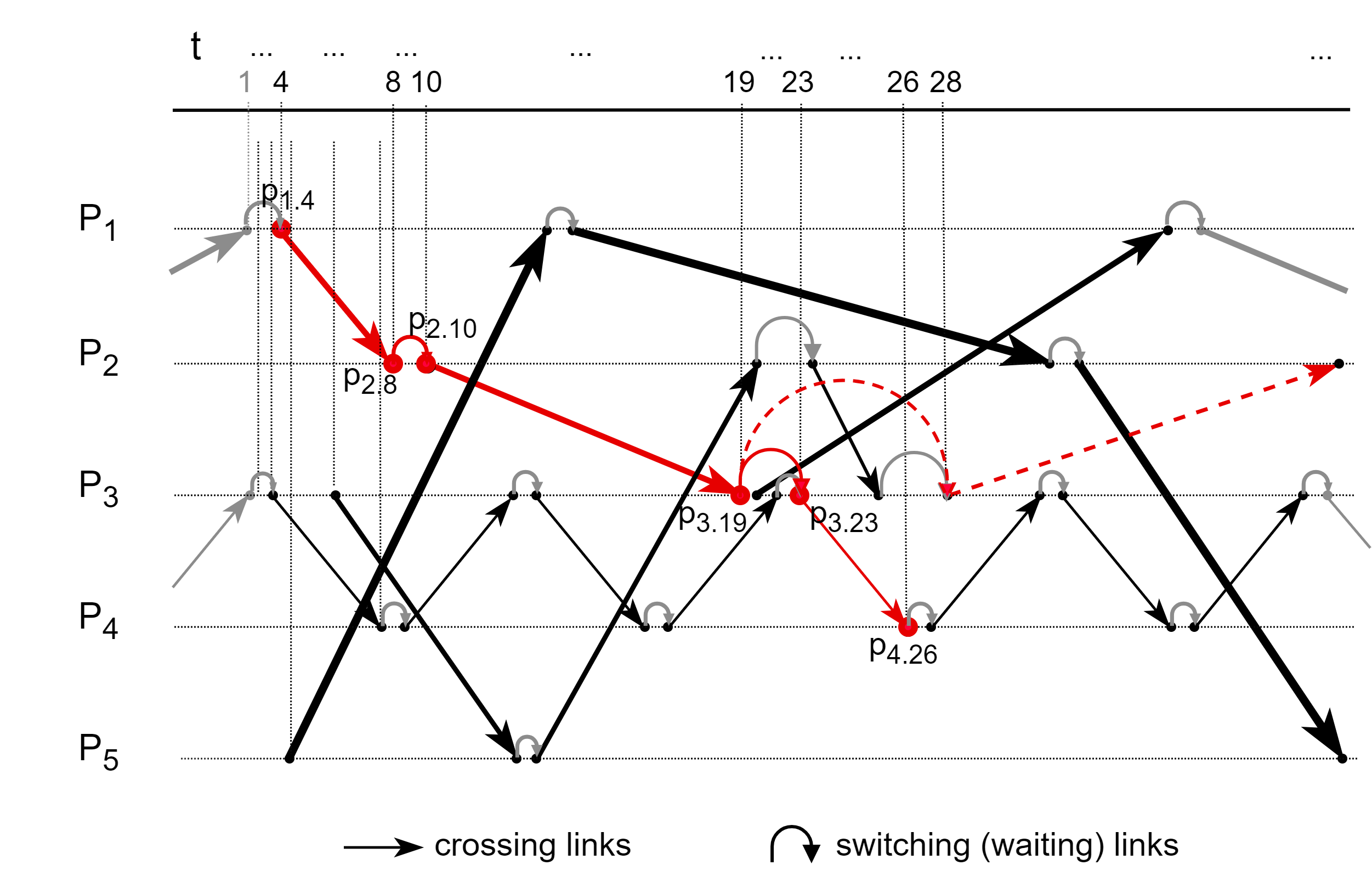

Supplement: Supplementary file 1 — Supplementary Information. [file 41598_2023_49723_MOESM1_ESM.zip › Timenet2.png]

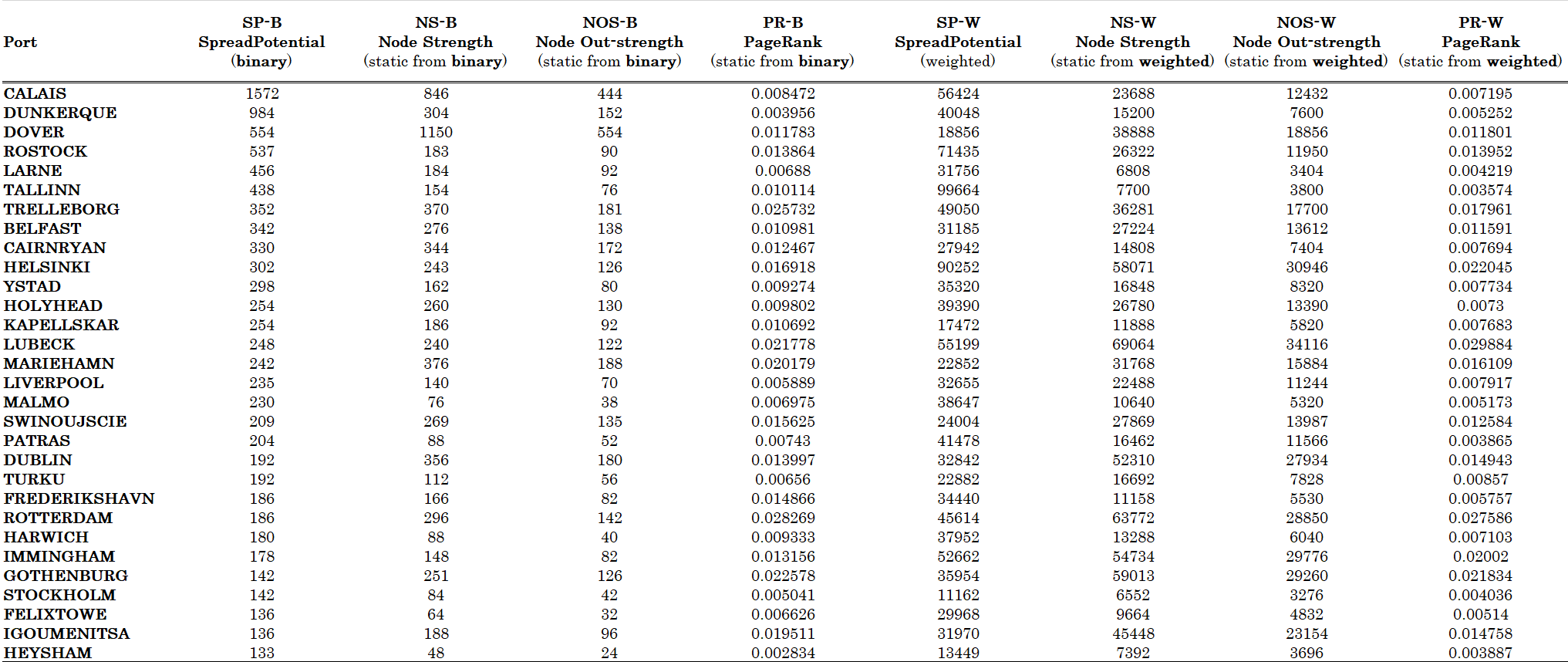

Supplement: Supplementary file 1 — Supplementary Information. [file 41598_2023_49723_MOESM1_ESM.zip › Ranking2.png]

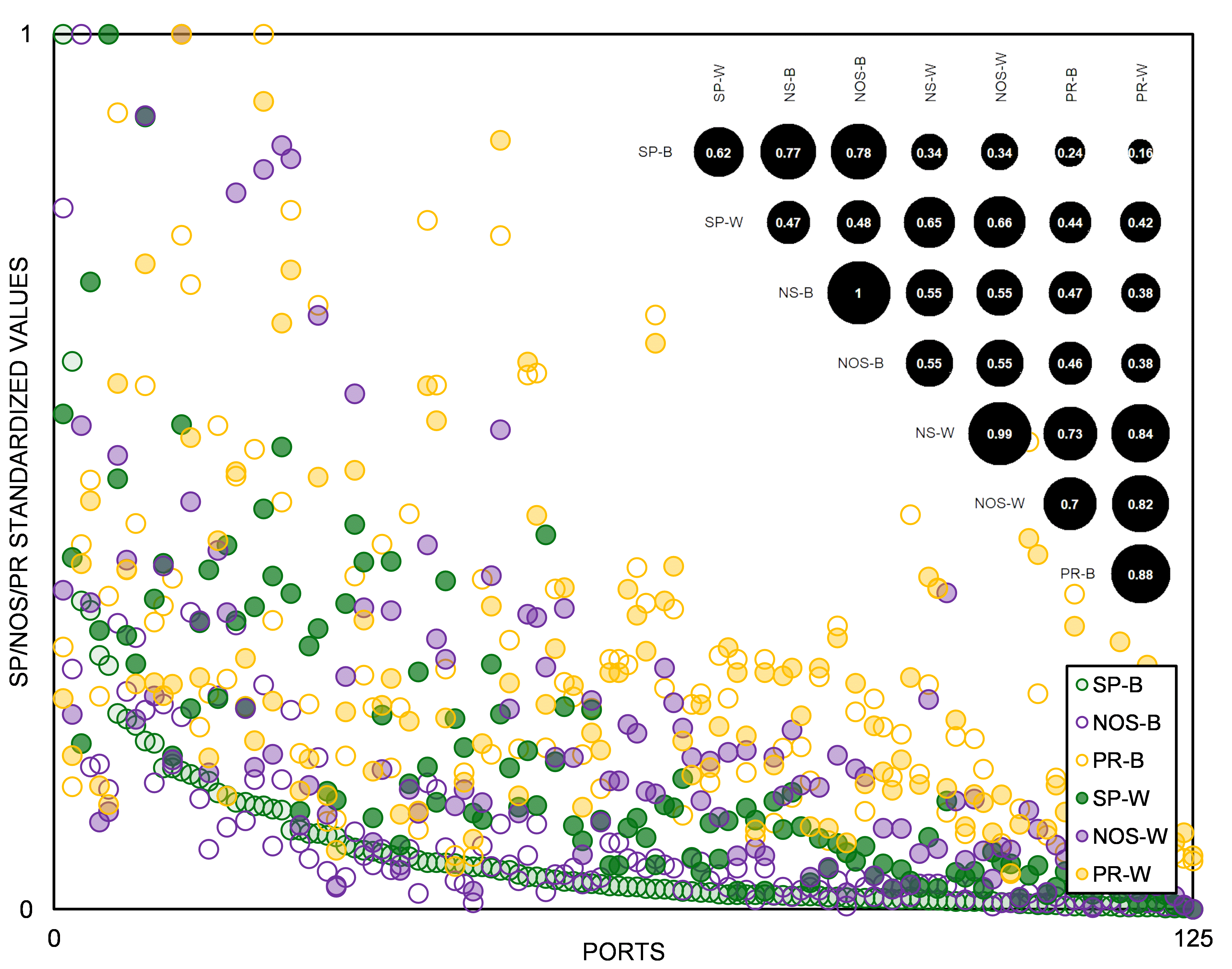

Supplement: Supplementary file 1 — Supplementary Information. [file 41598_2023_49723_MOESM1_ESM.zip › Correlations2.png]
